# A Polyclonal SELEX Aptamer Library Allows Differentiation of *Candida albicans*, *C. auris* and *C. parapsilosis* Cells from Human Dermal Fibroblasts

**DOI:** 10.3390/jof8080856

**Published:** 2022-08-15

**Authors:** Katharina Kneißle, Markus Krämer, Ann-Kathrin Kissmann, Hu Xing, Franziska Müller, Valerie Amann, Reiner Noschka, Kay-Eberhard Gottschalk, Anil Bozdogan, Jakob Andersson, Tanja Weil, Barbara Spellerberg, Steffen Stenger, Frank Rosenau

**Affiliations:** 1 Institute of Pharmaceutical Biotechnology, Ulm University, Albert-Einstein-Allee 11, 89081 Ulm, Germany; 2 Max Planck Institute for Polymer Research Mainz, Ackermannweg 10, 55128 Mainz, Germany; 3 Institute of Medical Microbiology and Hygiene, University Clinic of Ulm, Albert-Einstein-Allee 23, 89081 Ulm, Germany; 4 Institute of Experimental Physics, Ulm University, Albert-Einstein-Allee 11, 89081 Ulm, Germany; 5 Center for Electrochemical Surface Technology (CEST), Austrian Institute of Technology, 3420 Tulln, Austria; 6 AIT Austrian Institute of Technology, Biosensor Technologies, Giefinggasse 4, 1210 Vienna, Austria

**Keywords:** aptamer, biosensing, pathogenic yeasts, polyclonal library, SELEX

## Abstract

Easy and reliable identification of pathogenic species such as yeasts, emerging as problematic microbes originating from the genus *Candida*, is a task in the management and treatment of infections, especially in hospitals and other healthcare environments. Aptamers are seizing an already indispensable role in different sensing applications as binding entities with almost arbitrarily tunable specificities and optimizable affinities. Here, we describe a polyclonal SELEX library that not only can specifically recognize and fluorescently label *Candida* cells, but is also capable to differentiate *C. albicans*, *C. auris* and *C. parapsilosis* cells in flow-cytometry, fluorometric microtiter plate assays and fluorescence microscopy from human cells, exemplified here by human dermal fibroblasts. This offers the opportunity to develop diagnostic tools based on this library. Moreover, these specific and robust affinity molecules could also serve in the future as potent binding entities on biomaterials and as constituents of technical devices and will thus open avenues for the development of cost-effective and easily accessible next generations of electronic biosensors in clinical diagnostics and novel materials for the specific removal of pathogenic cells from human bio-samples.

## 1. Introduction

**Figure 1:**
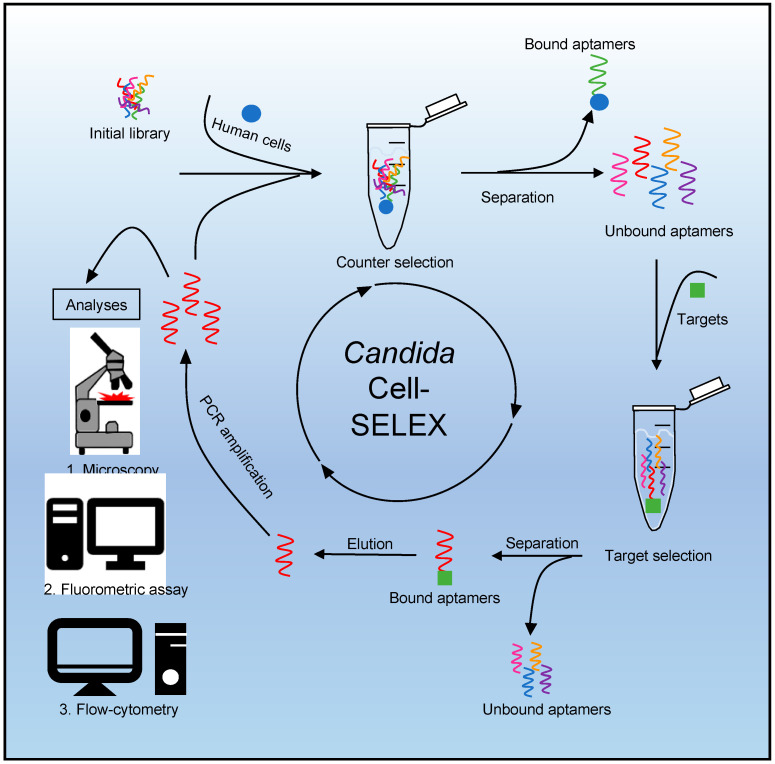
SELEX process and analytical techniques. Starting with a counter selection where an initial single-stranded (commercial) aptamer library (TriLink BioTechnologies, Inc, San Diego, CA, USA) with approximately 6 × 10^14^ individual molecules is incubated with human cells as the counter target. Bound aptamers can be removed by a washing step to exclude them from further selection. The focused library is used in a target selection by incubating it with target cells (*Candida*). Aptamers that do not bind to target cells are removed by washing. The aptamers specifically bound to the cells can now be eluted, amplified by polymerase chain reaction (PCR) using Cy5 and phosphate labeled primers and then subjected as single-stranded molecules to different analyses techniques after strand-separation, which is achieved by a strand digestion with lambda exonuclease. This process is repeated to obtain a specific polyclonal aptamer library for *Candida* after sufficient rounds of target binding and PCR-mediated amplification. Analytical techniques include 1. Fluorescence microscopy, 2. Fluorometric assay and 3. Flow-cytometry.

Since the introduction of aptamers more than 30 years ago, these single-stranded oligonucleotides (RNA or ssDNA) have emerged as serious alternatives to antibodies or antibody derivatives, offering significant additional technical options that make them increasingly attractive for various applications [1,2]. Aptamers are molecules with high specificity and affinity, surprising chemical and physical stability combined with a low overall immunogenicity. They can adopt different secondary and tertiary structures and serve as promising binding molecules in techniques requiring highly specific detection and quantification of target molecules [3]. In an iterative selection process performed completely in vitro, termed Systematic Evolution of Ligands by EXponential enrichment (SELEX), high-affinity aptamers can be evolved and isolated from large random sequence libraries (Figure 1). There aptamers can be selected against an enormous variety of different target structures ranging from small molecules such as metal ions [4], proteins [5], to cells and microorganisms [6,7,8,9] and even to complex targets such as cancerous tissues [10,11]. This directed selection process has continuously been improved and modified to create more powerful, robust and specialized strategies that allow the selection of aptamers with specific binding properties for different targets in numerous applications. Not only can they be used in various fields such as biomarker discovery [12], imaging agents [13], diagnostics [14], drug delivery [15] or as pharmaceutical compounds in molecular therapy [16,17], but they are also used as specific affinity molecules in the construction of binding units in technical devices [18] such as electronic biosensors [5,19], where mainly antibodies or their derivatives have been used [20,21]. Therefore, based on Cell-SELEX technology, the FluCell-SELEX has been developed in order to evolve aptamers to target intact living cells without knowing the exact membrane targets in advance [7,8,9]. We have recently shown that a major simplification of well-known aptamer isolation methods is the direct use of focused polyclonal libraries. Not only can they outperform single aptamers, as polyclonal libraries can be three orders of magnitude more sensitive in biosensing applications, but they are also anticipated to be beneficial for highly efficient target detection and improved performance due to the higher precision as well as the larger sequence space available [5,7]. This fast and reliable detection is of high importance, especially in the case of infections with pathogens such as bacteria or yeasts, in order to start immediate and appropriate treatments and to prevent a serious progression of the infection and the risk of septicemia. The majority of serious fungal infections are attributed to *Candida* species, as Candidiasis is the fourth most common cause of nosocomial infections with high mortality rates in systemic courses of 15–35% [22,23,24]. One entry site to the human body are wounds and wound care measures, since *Candida* has also been recognized as a problematic genus in wound infections in turn being a true challenge for wound management procedures [25,26,27,28]. However, most of the times *Candida* is not the typical pathogen for wound infections and mucosal membranes are often the entry point for *Candida* infections, since these yeasts can be found on mucous membranes as a classic colonizing flora [23]. The most commonly isolated species and the most widespread fungal pathogen worldwide is the yeast *Candida albicans* [29,30]. However, the leading role of *C. albicans* in invasive infections is decreasing and “non-albicans” *Candida* species such as *C. auris* or *C. parapsilosis* now account for a significant proportion of clinical isolates and their isolation rate has increased dramatically over the past 15 years [31]. The limited number of antifungals, the emergence of (multi-) drug resistances, the yeasts’ ability to form biofilms and the occurrence of misidentifications with other phylogenetically related pathogens have complicated the treatment of infections with *Candida* species [32,33,34,35]. Conventional identification methods of *Candida* rely on physiological or morphological attributes, but inadequacies of such techniques made them increasingly less reliable for precise and fast identification and differentiation [36]. Complex methods such as the matrix-assisted laser desorption/ionization time-of-flight mass spectrometry (MALDI-ToF) or commercially available identification systems, which are often based on the assimilation of carbon compounds, are promising in *Candida* differentiation; however, these diagnostic tools often rely on pure cultures, are not only considerably expensive, but also very time-consuming as it takes several days for results to be reported [37]. Correct and particularly rapid identification of pathogenic species such as yeasts from the genus *Candida* plays an important role in the management and in the overall outcome of an infection. Here, we describe a polyclonal SELEX library that not only can specifically recognize and fluorescently label *Candida*, but also is capable to discriminate between *C. albicans*, *C. auris*, *C. parapsilosis* cells and human cells here exemplified by human dermal fibroblasts (HDF). These specific and robust affinity molecules can serve as a potent binding entity and will open avenues for the development of cost-effective and easily accessible next generations of electronic biosensors in clinical diagnostics and novel materials for the specific removal of pathogenic cells from human bio-samples.

## 2. Materials and Methods

### 2.1. Cultivation of Cells

The yeast strains *C. albicans* (ATCC90028), *C. auris* (DSMZ-No. 21092), and *C. parapsilosis* (ATCC22019) were cultivated by inoculating a single colony in 5 mL YPD medium (1% *w*/*v* yeast extract, 2% *w*/*v* peptone, 2% *w*/*v* glucose), grown at 37 °C and orbital shaking at 150 rpm. HDF were cultivated in HDF medium (1% *v*/*v* Minimal Essential Medium Non-Essential Amino Acids, MEM NEAA), 1% *v*/*v* Penicillin/Streptavidin, 15% *v*/*v* Fetal Calf Serum (FCS) and 83 % *v*/*v* Dulbecco’s Modified Eagle Medium (DMEM) at 37 °C and 5% CO_2_. For testing with HDF cells, they were treated with accutase, counted with a Neubauer counting chamber and further 20,000 cells/well were cultivated in a 96 well plate for 24 h.

### 2.2. SELEX Procedure

SELEX was performed by a Cell-SELEX as described previously [7,9].

The first initial selection round was carried out with 1 nmol of synthesized and purified aptamer (TriLink BioTechnologies, Inc., San Diego, CA, USA) in phosphate-buffered saline (PBS) (137 mM NaCl, 10 mM Na_2_HPO_4_, 2.7 mM KCl and 1.8 mM KH_2_PO_4_, pH 7.4). For all following SELEX rounds 1.5 pmol aptamer was used. The activation of the aptamers occurred as follows: 5 min at 95 °C, 5 min at 4 °C, and 20 min at 25 °C. From round 1 to round 5 individual aptamers for each *Candida* were prepared. For this, the counter selection of each *Candida* aptamers was composed out of the two other *Candida* (250 µL with an O.D._600_ of 1), the selection round with the target *Candida* (250 µL, O.D._600_ of 1). Additionally, from round 1 on, the concentration of added tRNA (10 mg/mL) (Thermo Fisher Scientific, Waltham, Massachusetts, USA) and bovine serum albumin (BSA) (100 mg/mL) (Carl Roth, Karlsruhe, Germany) during the selection round were increased as well as the number of washing steps until round 5 (exact information see Table 1). From round 6 on, the individual aptamers were pooled, as counter selection target HDF cells (20,000 cells/well) were used.

Between the individual SELEX rounds the eluted aptamers were resubjected into the selection process after PCR-mediated amplification and separation of the unwanted reverse DNA strand. Therefore, a Cy5-labeled forward primer (sequence: 5′-[Cy5]-TAGGGAAGAGAAGGACATATGAT) and a phosphate labeled reverse primer (sequence: 5′-[Phosphate]-TCAAGTGGTCATGTACTAGTCAA) (Eurofins Genomics, Ebersberg, Germany) were used.

For the following strand separation, the PCR product was purified by purification kit (Qiagen, Hilden, Germany) by using 3 volumes of buffer NTI and 5 volumes of isopropanol. For generating ssDNA, a digestion with Lambda Exonuclease (Thermo Fisher Scientific, Waltham, MA, USA), which degrades 5′ phosphorylated linear dsDNA from 5′ to 3′ direction, was conducted. After purification, the dsDNA was incubated with lambda exonuclease for 30 min at 37 °C and 150 rpm. The inactivation of the exonuclease was performed at 80 °C for 10 min. Finally, the ssDNA was also purified by the purification kit using 2 volumes of NTC buffer and 3 volumes of isopropanol.

### 2.3. Real-Time PCR

For further analysis of the specific aptamer library a quantitative PCR (qPCR) was performed with the eluates of the SELEX rounds six to eight using SYBR green dye (final concentration 0.5×) (Sigma-Aldrich, St. Louis, MO, USA) and qTOWER^3^G touch (Analytik Jena GmbH, Jena, Germany). A standard curve in the range of 0.01–1 ng was prepared with a synthetic aptamer library. Afterwards, a melting curve analysis and the determination of the Ct value could be conducted from which the DNA quantity in the sample could be calculated.

### 2.4. Analyses of Specific Aptamer Library

#### 2.4.1. Fluorometric Assay

To determine the specific binding of the aptamer library a fluorometric assay was performed. Therefore, 10 pmol of fluorescent labeled aptamers with Cy5 (excitation 635 nm and emission 670 nm) were activated. A total of 20,000 *Candida* cells were prepared by centrifugation at 11,000× *g* and washed 1 time with PBS. Additionally, the adherent HDF cells were rinsed once with PBS. Activated aptamer solution was added to the *Candida* pellet or adherent HDF and incubated for 1 h at 37 °C (and 5 % CO_2_ for HDF). After incubation the supernatant of HDF cells was discarded, accutase was added and incubated for approximately 20 min. Thereafter, HDF cells were centrifuged for 3 min at 2000 rpm. *Candida* were also centrifuged after incubation at 9000× *g* for 3 min and the pellets were resuspended in 100 µL PBS. Elution of the bound aptamers was obtained by heating to 95 °C for 5 min followed by another centrifugation step for HDF (2000× *g*, 3 min) and *Candida* (9000× *g*, 3 min).

For measuring of the fluorescent eluted aptamers, a Tecan infinite M200 microplate reader (Tecan group AG, Männedorf, Switzerland) with an excitation wavelength of 635 nm and an emission wavelength of 670 nm was used.

#### 2.4.2. Microscopy

To further illustrate the specific aptamer binding, the cells were microscopically analyzed using a Leica DMi8 coded (Leica Microsystems CMS GmbH, Wetzlar, Germany). For this purpose, 5 pmol of the specific aptamer library was activated. Furthermore, 20,000 cells each of *C. albicans, C. auris, C. parapsilosis* and *Candida* were prepared by centrifugation and a washing step with PBS. In addition, 20,000 HDF cells per well were left adhered and rinsed once with PBS.

First, all cells were incubated individually with the aptamer solution for 15 min at 37 °C. Incubation was followed by centrifugation at 9000× *g* for 3 min and a wash step with PBS for the individual experiments of the different *Candida*. The HDF cells were rinsed with 250 µL.

In further experiments, the individual *Candida* and a *Candida* mix were each incubated with HDF cells for 90 min, followed by careful rinsing with PBS. Subsequently, the cell mix was incubated with already activated aptamers for 15 min, followed by a washing step with PBS.

For fluorescence microscopy, cells were resuspended in 100 µL PBS and placed on a 96 well plate.

#### 2.4.3. Flow-Cytometry

To further test the binding specificity of the aptamer library, flow-cytometry experiments were performed using a FACScalibur and Flowjo (BD, Franklin Lakes, NJ, USA). For this purpose, the cells were prepared as described above (2.4.2 Microscopy). *Candida* and HDF were tested individually each with 10 pmol aptamer.

Incubation was followed by centrifugation at 9000× *g* and 3 min for *Candida*, 2000× *g* and 3 min for HDF. Pellets were resuspended in 300 µL PBS and transferred to flow-cytometry tubes. For flow-cytometry, 10,000 cells were measured.

## 3. Results

**Figure 2:**
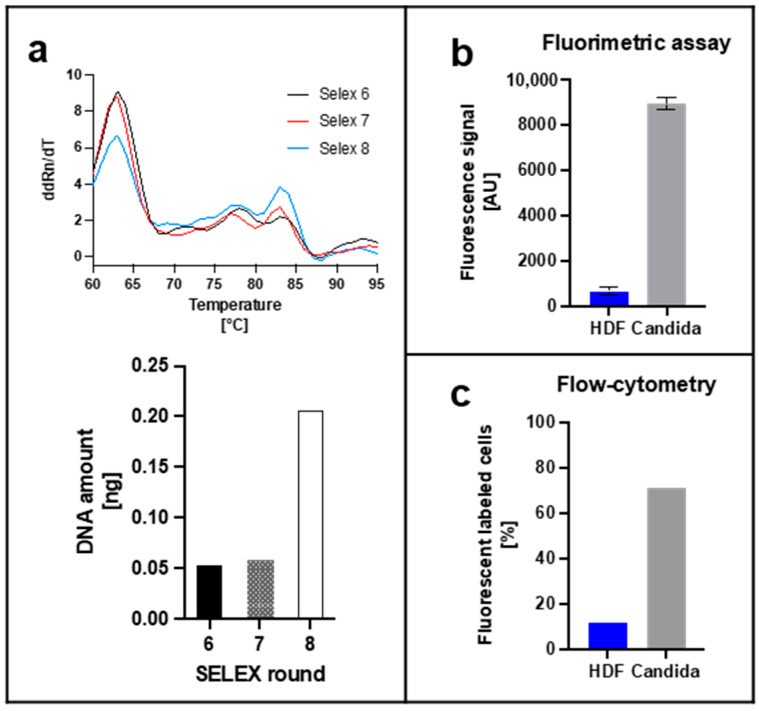
Evolution and specific labeling of mixed *Candida* cells by the SELEX-derived aptamer library R8. (**a**) Real-time PCR based analysis of melting curves of the round six to eight aptamer libraries (upper panel) and DNA concentrations in eluates immediately after the SELEX rounds (lower panel). P1 and P2 are the two major temperature peaks observed in the experiments. (**b**) Identification of *Candida* by fluorometric assays. HDF cells and *Candida* were incubated with aptamer and compared by fluorescence measurement. (**c**) Specific identification of *Candida* by flow cytometry. Incubation of cells together with aptamer followed by flow-cytometry analysis.

Based on the previous experience with bacterial whole cell processes performed in our laboratory, the *Candida* SELEX was performed for eight rounds of selection and amplification with increasingly harsh elimination conditions for a fast development of binding specificity. During a SELEX process the respective libraries of each round run through an evolutionary process with characteristic consequences for the sequence space and the composition of the individual sequences. Typically, a bias is introduced towards higher GC-content of the molecules [7,9]. This is accompanied by an increase in melting temperatures for the higher GC-content aptamers in PCR reactions. We tentatively tested this for the final rounds six to eight and found that in fact two major peaks occurred for the melting temperatures at 63 °C and 83 °C (peak 1 and peak 2; P1 and P2 in Figure 2a) in the real-time PCR analysis (Figure 2a). Between round six and eight, the relative fluorescence intensity (ddRn/dT-value) dropped for peak 1 and simultaneously increased for peak 2 indicating the expected shift. The real-time PCR analysis revealed that the final DNA concentration of the eluted focused aptamer libraries immediately after the SELEX rounds was highest in round eight. Subsequently, the resulting round 8 library R8 was further characterized by fluorometric assays with cells submerged in liquid media, flow-cytometric analyses using the library for fluorescent labelling and in fluorescence microscopy. The aim was to demonstrate the desired ability of R8 to allow differential staining of *Candida* cells to distinguish them from human cells exemplified in our experiments by human dermal fibroblast, a minimalized set-up resembling a model of early stage wound infections by the pathogenic yeasts. Upon labelling of planktonic cells from liquid cultures *Candida* was clearly distinguishable from HDF cells in the fluorometric assay (Figure 2b). The same was true in the flow-cytometric measurements of samples containing adjusted equal cell numbers of either HDF or *Candida* cells, in which on the single cell level approximately 75% of the *Candida* cells were bound by aptamers (Figure 2c and Figure S1).

**Figure 3:**
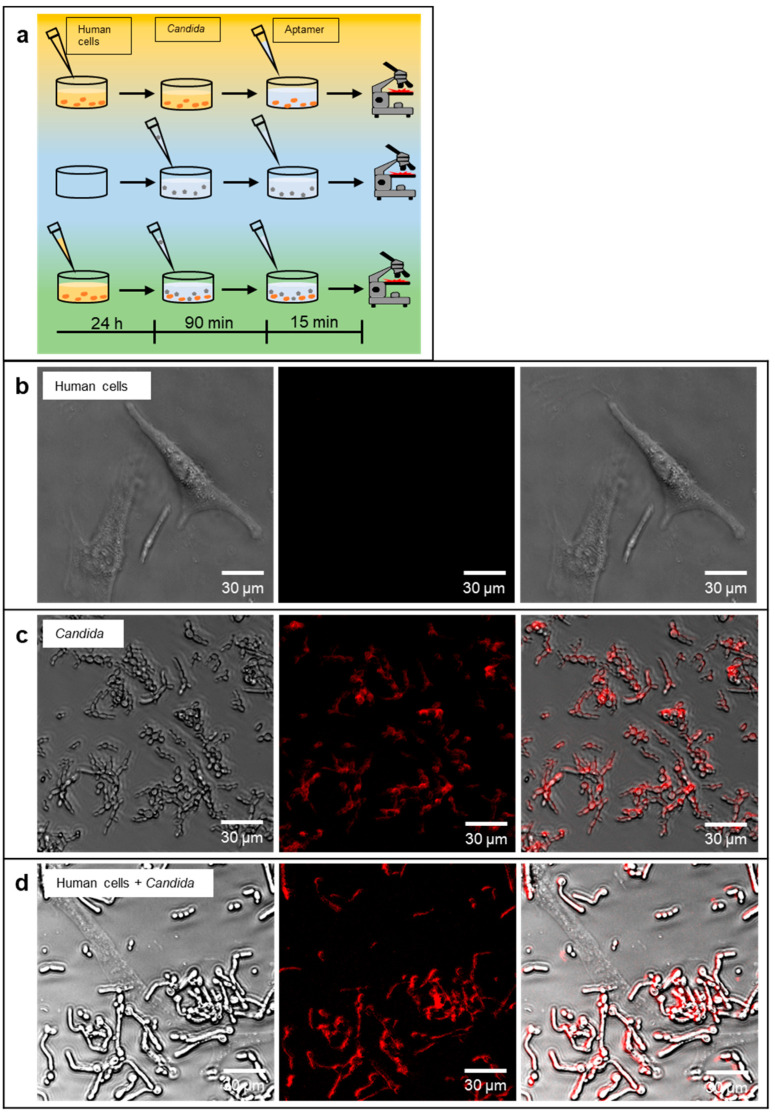
Specific labeling of mixed *Candida* cells by the SELEX-derived aptamer library R8. (**a**) Experimental set-up of the microscopic differentiation analyses for *Candida* and human cells (HDF). Incubation of single cells individually and as a mixture together with aptamer. (**b**) Microscopic images of human cells with aptamer as the negative control (transmitted light, fluorescence, merge). (**c**) *Candida* alone as the positive control. (**d**) Fluorescence micrographs of a mixture of human cells “infected” with *Candida*. *Candida* cells were allowed to bind to the substratum of the cell culture flask including the human cells for 90 min prior the analysis.

**Figure 4:**
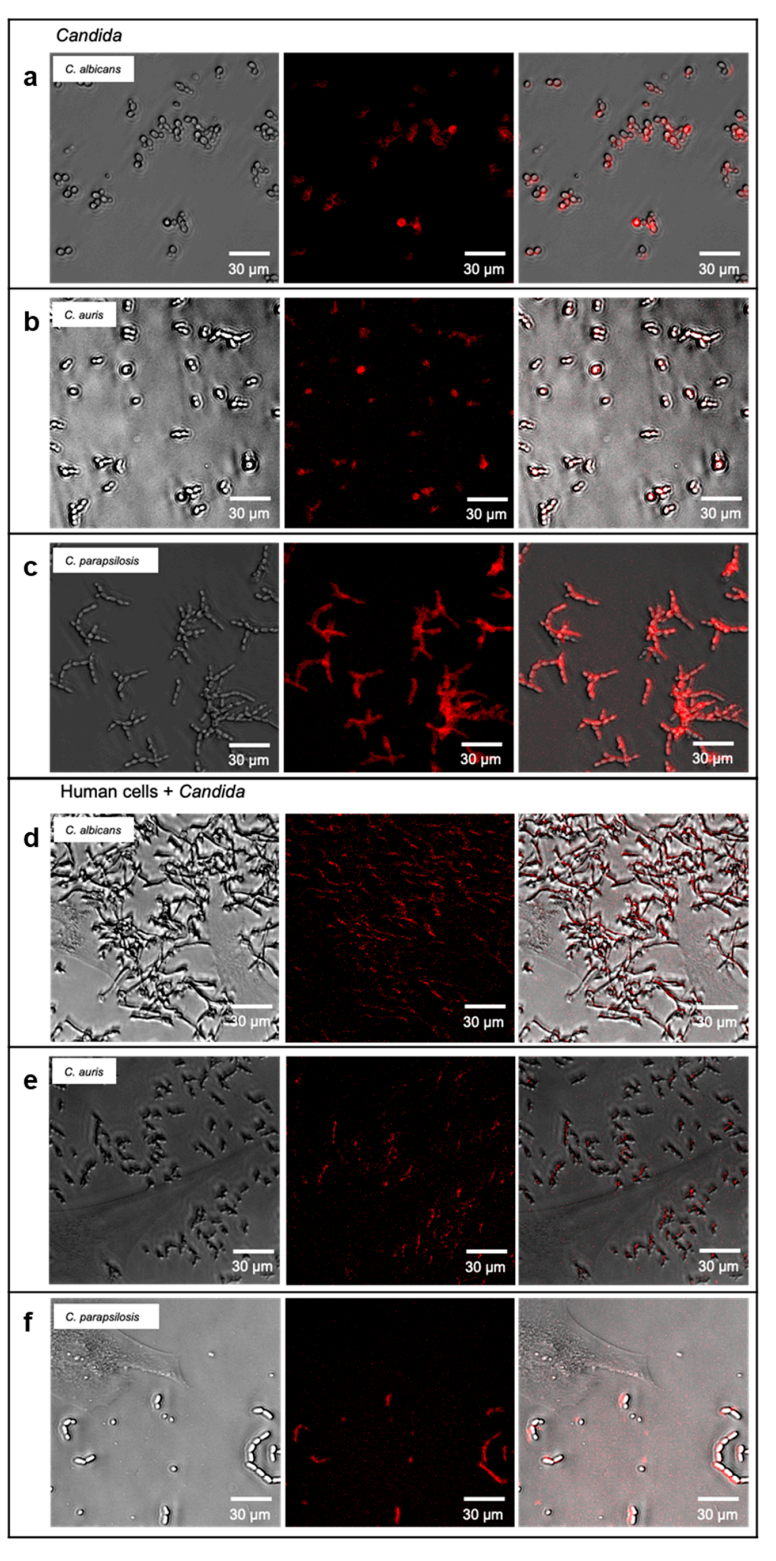
Specific labeling of individual *Candida* cells by the SELEX-derived aptamer library R8. Experimental set-up was as in Figure 3b–d with the difference that individual *Candida* species were used instead of mixes. Panels (**a**–**c**): *Candida* species alone as positive controls. Panels (**d**–**f**): *Candida* species on human cells.

The major intention of this initial study on anti-*Candida* aptamers for future sensing applications was to evaluate, whether a polyclonal library was already suited to allow specific identification of *Candida* cells in different detection techniques widely used for microbes. A second important property would be the ability to label the target cells for fluorescence microscopy to allow differential staining of *Candida* and human cells alone and the yeast cells preincubated with the HDF cells prior to microscopic analysis, an experiment, which was intended to mimic a very early stage of a wound infection (Figure 3a). We thus tested this ability in first instance with the mix of *Candida* cells in comparison to the human cells. Whereas in the control experiment the aptamer library completely failed to label HDF cells, *Candia* cells could be visualized by this technique (Figure 3b,c). It was also possible to stain *Candida* cells, which were allowed to adhere to the human cells for 90 min before the staining with the polyclonal aptamer library R8. As intended, HDF cells were also not stained in this preincubation/infection experiment and the R8 library proved in this initial set of experiments that selective staining was possible (Figure 3d). Using the individual species *C. albicans*, *C. auris* and *C. parapsilosis* instead of the mixed species samples, it was also possible to collocate fluorescence and cells as before in the microscopic method, when pure *Candida* cells were analyzed (Figure 4a–c). The same was possible in the preincubation model in presence of HDF cells, which were not stained in contrast to cells of the three individual *Candida* species which could be differentiated from the human cells in these experiments (Figure 4d–f).

## 4. Discussion

The development of novel analytical techniques including the construction of sensor chips requires—if the concept is based on specific recognition and binding of the desired analyte—the selection of the appropriate binding molecules, which in the case of aptamers originate from molecular evolution in one of the variform SELEX processes existing today [6,38]. Breaking the habituality aptamer research to isolate individual aptamers from SELEX libraries and to not declare a project finished before this, which is per se more than reasonable, in recent years, it became apparent that biotechnological applications cannot only work already directly after SELEX, but also that these polyclonal libraries can be more efficient in detection methods [7,9]. The SELEX evolutionary processes successfully delivering functional binding molecules as binding entities on a sensor chip thus represents a first important, if not the most relevant technological achievement in the development of a biosensor chip [5]. This evolution process was monitored using real-time PCR analyses where a shift towards higher melting temperatures was observed. This indicates that the aptamer library R8 shows an elevated GC content, which is indicative for a successful evolution process resulting in drastically different sequences in the final round. Whole cell SELEX as a concept has proven its enormous potential to isolate aptamers specifically binding one or, in the case of polyclonal libraries, manifold individual cell surface structures, with the latter strategy contingently being more performant and robust in the detection of different variants of the target cells [7]. The polyclonal library against *Candida* cells generated in the study presented here as a short communication represents the first preliminary but important technological achievement on the way to novel diagnostic techniques for these increasingly important pathogenic yeasts. As expected, based on our experience and the stringency of selection (including counter selection against human cells) after eight rounds of SELEX a considerable specificity was achieved against the *Candida* cell samples which could clearly be distinguished from human HDF model cells in the fluorometric assay system. On the single cell level, in the flow-cytometric analysis with more than 75% the majority of individual *Candida* cells were labeled using the initial and tentative experimental protocol for the labeling process. Microbial cells including *Candida* pass through major physiological modification processes while living in the different growth phases [39,40,41,42,43]. A polyclonal library against carbapenem-resistant *Pseudomonas aeruginosa* was shown to be sensitive towards such differences of the cell wall between cells originating from different growth phases; nevertheless, it was more performant concerning the identification of a larger set of clinical isolates compared to individual aptamers [7]. Without having attempted further optimization of the libraries or isolation of individual aptamers library R8 was functional in labeling the target cells, in mixes as well as individual strains, and thus represents a robust fundament for improving the uniform labeling of *Candida* cells in flow-cytometry. Nevertheless, the existing R8 library was fully functional for specific labeling of *Candida* cells to clearly distinguish them from human cells in fluorescence microscopy. Although representing a drastic simplification, our initial experiments presented here mimic an early stage of a *Candida*-infection as it can occur in hospitals in patients with skin defects as burn wounds or after surgery. In this set-up, it was possible to identify the pathogen in the background of human cells which demonstrates that already R8 allows diagnostic applications. Aptamers as binding entities can also be used for the easy functionalization of a variety of materials including polymers and hydrogels. We have recently shown that aptamer functionalized protein hydrogel microbeads can serve as affinity materials for binding and enrichment of pathogenic bacteria from human serum and blood [18]. Biomaterials combined with affinity molecules can also serve as complex next-generation wound dressings [28,44] and it appears obvious that also aptamers or aptamer libraries as presented here against pathogenic microbes can be used as affinity determining constituents of such medical materials. The same is true for the construction of electronic biosensors dedicated to the sensitive quantification of pathogens in human samples such as blood of patients suspected to suffer from septicemia. With the initial but important results presented in this study, we hope to inspire other researchers to create applications based on the R8 or similar pathogen dedicated libraries and we believe that our goal to develop both, biomaterials equipped with affinity to bind different *Candida*-species for medical applications and a generation of *Candida*-specific gFET devices, can be approached based on our results.

## 5. Conclusions

A polyclonal aptamer library has been developed which allows the identification of *Candida*-species either in monoculture or mixed culture. For method-establishing the strains *C. albicans*, *C. auris* and *C. parapsilosis*. The aptamer library was functional in standard diagnostic techniques as flow-cytometry, fluorometric microtiter plate assays and fluorescence microscopy.

## Figures and Tables

**Table 1:** Overview of SELEX round 1 to 8 with used tRNA and BSA concentrations during target selection round and washing steps after target selection round. Round 1 to 5 were individual aptamers for *C. albicans*, *C. auris* and *C. parapsilosis*, from round 6 on, HDF cells were used for counter selection, *Candida* mixture for target selection.

SELEX Round	tRNA and BSA [pmol]	Washing Steps with PBS	HDF Counter-Selection
1	600	1	-
2	900	2	-
3	1500	4	-
4	2100	6 (4–6 with 0.1% (*w*/*v*) BSA in PBS)	-
5	2600	8 (5–8 with 0.1% (*w*/*v*) BSA in PBS)	-
6	3000	8 (5–8 with 0.1% (*w*/*v*) BSA in PBS)	+
7	3000	8 (5–8 with 0.1% (*w*/*v*) BSA in PBS)	+
8	3000	8 (5–8 with 0.1% (*w*/*v*) BSA in PBS)	+

## Data Availability

Not applicable.

## Supplementary Materials

The following supporting information can be downloaded at: https://www.mdpi.com/article/10.3390/jof8080856/s1, Figure S1: Flow-cytometry data show detection of fluorescently labeled *Candida* cells.

## References

[1] Ellington A.D., Szostak J.W. (1990). In Vitro Selection of RNA Molecules That Bind Specific Ligands. Nature.

[2] Tuerk C., Gold L. (1990). Systematic Evolution of Ligands by Exponential Enrichment: RNA Ligands to Bacteriophage T4 DNA Polymerase. Science.

[3] Sharma T.K., Bruno J.G., Dhiman A. (2017). ABCs of DNA Aptamer and Related Assay Development. Biotechnol. Adv..

[4] Qu H., Csordas A.T., Wang J., Oh S.S., Eisenstein M.S., Soh H.T. (2016). Rapid and Label-Free Strategy to Isolate Aptamers for Metal Ions. ACS Nano.

[5] Kissmann A.-K., Andersson J., Bozdogan A., Amann V., Kraemer M., Xing H., Raber H., Kubiczek D.H., Aspermair P., Knoll W. (2022). Polyclonal Aptamer Libraries as Binding Entities on a Graphene FET Based Biosensor for the Discrimination of Apo- and Holo- Retinol Binding Protein 4. Nanoscale Horiz..

[6] Kubiczek D., Bodenberger N., Rosenau F. (2017). Aptamers as Promising Agents in Diagnostic and Therapeutic Applications. Antimicrob. Res. Nov. Bioknowl. Educ. Programs.

[7] Kubiczek D., Raber H., Bodenberger N., Oswald T., Sahan M., Mayer D., Wiese S., Stenger S., Weil T., Rosenau F. (2020). The Diversity of a Polyclonal FluCell-SELEX Library Outperforms Individual Aptamers as Emerging Diagnostic Tools for the Identification of Carbapenem Resistant *Pseudomonas aeruginosa*. Chem.–A Eur. J..

[8] Xing H., Kissmann A.K., Raber H.F., Krämer M., Amann V., Kohn K., Weil T., Rosenau F. (2021). Polyclonal Aptamers for Specific Fluorescence Labeling and Quantification of the Health Relevant Human Gut Bacterium Parabacteroides Distasonis. Microorganisms.

[9] Raber H.F., Kubiczek D.H., Bodenberger N., Kissmann A.K., D’souza D., Xing H., Mayer D., Xu P., Knippschild U., Spellerberg B. (2021). Flucell-selex Aptamers as Specific Binding Molecules for Diagnostics of the Health Relevant Gut Bacterium Akkermansia Muciniphila. Int. J. Mol. Sci..

[10] Zhong W., Pu Y., Tan W., Liu J., Liao J., Liu B., Chen K., Yu B., Hu Y., Deng Y. (2019). Identification and Application of an Aptamer Targeting Papillary Thyroid Carcinoma Using Tissue-SELEX. Anal. Chem..

[11] Li L., Wan J., Wen X., Guo Q., Jiang H., Wang J., Ren Y., Wang K. (2021). Identification of a New DNA Aptamer by Tissue-SELEX for Cancer Recognition and Imaging. Anal. Chem..

[12] Minopoli A., Della Ventura B., Lenyk B., Gentile F., Tanner J.A., Offenhäusser A., Mayer D., Velotta R. (2020). Ultrasensitive Antibody-Aptamer Plasmonic Biosensor for Malaria Biomarker Detection in Whole Blood. Nat. Commun..

[13] Zhang J., Smaga L.P., Satyavolu N.S.R., Chan J., Lu Y. (2017). DNA Aptamer-Based Activatable Probes for Photoacoustic Imaging in Living Mice. J. Am. Chem. Soc..

[14] Sekhon S.S., Kaur P., Kim Y.H., Sekhon S.S. (2021). 2D Graphene Oxide–Aptamer Conjugate Materials for Cancer Diagnosis. Npj 2d Mater. Appl..

[15] Liang T., Yao Z., Ding J., Min Q., Jiang L., Zhu J.J. (2018). Cascaded Aptamers-Governed Multistage Drug-Delivery System Based on Biodegradable Envelope-Type Nanovehicle for Targeted Therapy of HER2-Overexpressing Breast Cancer. ACS Appl. Mater. Interfaces.

[16] Zhou J., Rossi J. (2017). Aptamers as Targeted Therapeutics: Current Potential and Challenges. Nat. Rev. Drug Discov..

[17] Mahlknecht G., Maron R., Mancini M., Schechter B., Sela M., Yarden Y. (2013). Aptamer to ErbB-2/HER2 Enhances Degradation of the Target and Inhibits Tumorigenic Growth. Proc. Natl. Acad. Sci. USA.

[18] Krämer M., Kissmann A.-K., Raber H.F., Xing H., Favella P., Müller I., Spellerberg B., Weil T., Kubiczek D., Sihler S. (2021). BSA Hydrogel Beads Functionalized with a Specific Aptamer Library for Capturing Pseudomonas Aeruginosa in Serum and Blood. Int. J. Mol. Sci..

[19] Aspermair P., Mishyn V., Bintinger J., Happy H., Bagga K., Subramanian P., Knoll W., Boukherroub R., Szunerits S. (2021). Reduced Graphene Oxide–Based Field Effect Transistors for the Detection of E7 Protein of Human Papillomavirus in Saliva. Anal. Bioanal. Chem..

[20] Afsahi S., Lerner M.B., Goldstein J.M., Lee J., Tang X., Bagarozzi D.A., Pan D., Locascio L., Walker A., Barron F. (2018). Novel Graphene-Based Biosensor for Early Detection of Zika Virus Infection. Biosens. Bioelectron..

[21] Seo G., Lee G., Kim M.J., Baek S.-H., Choi M., Ku K.B., Lee C.-S., Jun S., Park D., Kim H.G. (2020). Rapid Detection of COVID-19 Causative Virus (SARS-CoV-2) in Human Nasopharyngeal Swab Specimens Using Field-Effect Transistor-Based Biosensor. ACS Nano.

[22] Pfaller M.A., Diekema D.J. (2007). Epidemiology of Invasive Candidiasis: A Persistent Public Health Problem. Clin. Microbiol. Rev..

[23] Lockhart S.R. (2014). Current Epidemiology of Candida Infection. Clin. Microbiol. Newsl..

[24] Quindós G., Marcos-Arias C., San-Millán R., Mateo E., Eraso E. (2018). The Continuous Changes in the Aetiology and Epidemiology of Invasive Candidiasis: From Familiar Candida Albicans to Multiresistant Candida Auris. Int. Microbiol. Off. J. Span. Soc. Microbiol..

[25] Kalan L., Grice E.A. (2018). Fungi in the Wound Microbiome. Adv. Wound Care.

[26] Amann V., Kissmann A.-K., Krämer M., Krebs I., Perez-Erviti J.A., Otero-Gonzalez A.J., Morales-Vicente F., Rodríguez A., Ständker L., Weil T. (2022). Increased Activities against Biofilms of the Pathogenic Yeast Candida Albicans of Optimized Pom-1 Derivatives. Pharmaceutics.

[27] Kubiczek D., Raber H., Gonzalez-García M., Morales-Vicente F., Staendker L., Otero-Gonzalez A.J., Rosenau F. (2020). Derivates of the Antifungal Peptide Cm-P5 Inhibit Development of Candida Auris Biofilms In Vitro. Antibiotics.

[28] Kubiczek D., Flaig C., Raber H., Dietz S., Kissmann A., Heerde T., Bodenberger N., Wittgens A., González-Garcia M., Kang F. (2020). A Cerberus-Inspired Anti-Infective Multicomponent Gatekeeper Hydrogel against Infections with the Emerging “Superbug” Yeast *Candida auris*. Macromol. Biosci..

[29] Papon N., Courdavault V., Clastre M., Bennett R.J. (2013). Emerging and Emerged Pathogenic Candida Species: Beyond the Candida Albicans Paradigm. PLoS Pathog..

[30] Noble S.M., French S., Kohn L.A., Chen V., Johnson A.D. (2010). Systematic Screens of a Candida Albicans Homozygous Deletion Library Decouple Morphogenetic Switching and Pathogenicity. Nat. Genet..

[31] Pfaller M.A., Diekema D.J., Gibbs D.L., Newell V.A., Ellis D., Tullio V., Rodloff A., Fu W., Ling T.A. (2010). Results from the ARTEMIS DISK Global Antifungal Surveillance Study, 1997 to 2007: A 10.5-Year Analysis of Susceptibilities of Candida Species to Fluconazole and Voriconazole as Determined by CLSI Standardized Disk Diffusion. J. Clin. Microbiol..

[32] Černáková L., Roudbary M., Brás S., Tafaj S., Rodrigues C.F. (2021). Candida Auris: A Quick Review on Identification, Current Treatments, and Challenges. Int. J. Mol. Sci..

[33] Rajendran R., Sherry L., Nile C.J., Sherriff A., Johnson E.M., Hanson M.F., Williams C., Munro C.A., Jones B.J., Ramage G. (2016). Biofilm Formation Is a Risk Factor for Mortality in Patients with Candida Albicans Bloodstream Infection-Scotland, 2012–2013. Clin. Microbiol. Infect..

[34] Enoch D.A., Yang H., Aliyu S.H., Micallef C. (2017). The Changing Epidemiology of Invasive Fungal Infections. Methods Mol. Biol..

[35] Morales-López S.E., Parra-Giraldo C.M., Ceballos-Garzón A., Martínez H.P., Rodríguez G.J., Álvarez-Moreno C.A., Rodríguez J.Y. (2017). Invasive Infections with Multidrug-Resistant Yeast Candida Auris, Colombia. Emerg. Infect. Dis..

[36] Neppelenbroek K., Seó R., Urban V., Silva S., Dovigo L., Jorge J., Campanha N. (2014). Identification of Candida Species in the Clinical Laboratory: A Review of Conventional, Commercial, and Molecular Techniques. Oral Dis..

[37] Jamal W.Y., Ahmad S., Khan Z.U., Rotimi V.O. (2014). Comparative Evaluation of Two Matrix-Assisted Laser Desorption/Ionization Time-of-Flight Mass Spectrometry (MALDI-TOF MS) Systems for the Identification of Clinically Significant Yeasts. Int. J. Infect. Dis..

[38] Zhuo Z., Yu Y., Wang M., Li J., Zhang Z., Liu J., Wu X., Lu A., Zhang G., Zhang B. (2017). Recent Advances in SELEX Technology and Aptamer Applications in Biomedicine. Int. J. Mol. Sci..

[39] Bravo Ruiz G., Ross Z.K., Gow N.A.R., Lorenz A. (2020). Pseudohyphal Growth of the Emerging Pathogen Candida Auris Is Triggered by Genotoxic Stress through the S Phase Checkpoint. Msphere.

[40] Chen H., Zhou X., Ren B., Cheng L. (2020). The Regulation of Hyphae Growth in *Candida albicans*. Virulence.

[41] Chen C., Zeng G., Wang Y. (2018). G1 and S Phase Arrest in *Candida Albicans* Induces Filamentous Growth via Distinct Mechanisms. Mol. Microbiol..

[42] Gow N.A. (1997). Germ Tube Growth of Candida Albicans. Curr. Top. Med. Mycol..

[43] Ogasawara A., Odahara K., Toume M., Watanabe T., Mikami T., Matsumoto T. (2006). Change in the Respiration System of Candida Albicans in the Lag and Log Growth Phase. Biol. Pharm. Bull..

[44] Bodenberger N., Kubiczek D., Halbgebauer D., Rimola V., Wiese S., Mayer D., Rodriguez Alfonso A.A., Ständker L., Stenger S., Rosenau F. (2018). Lectin-Functionalized Composite Hydrogels for “Capture-and-Killing” of Carbapenem-Resistant *Pseudomonas aeruginosa*. Biomacromolecules.

