# Supplementary material for: A Polyclonal SELEX Aptamer Library Allows Differentiation of Candida albicans, C. auris and C. parapsilosis Cells from Human Dermal Fibroblasts
Source: J Fungi (Basel). 2022 Aug 15;8(8):856. doi: 10.3390/jof8080856 (PMC9410195; doi:10.3390/jof8080856)
Supplement: Supplementary file 1 [file jof-08-00856-s001.zip › jof-1838930 - supplementary.pdf]

Journal of *Fungi*

MDPI

A Polyclonal SELEX Aptamer Library Allows Differentiation of *Candida albicans, C. auris* and *C. parapsilosis* Cells from Human Dermal Fibroblasts

Supplementary Materials

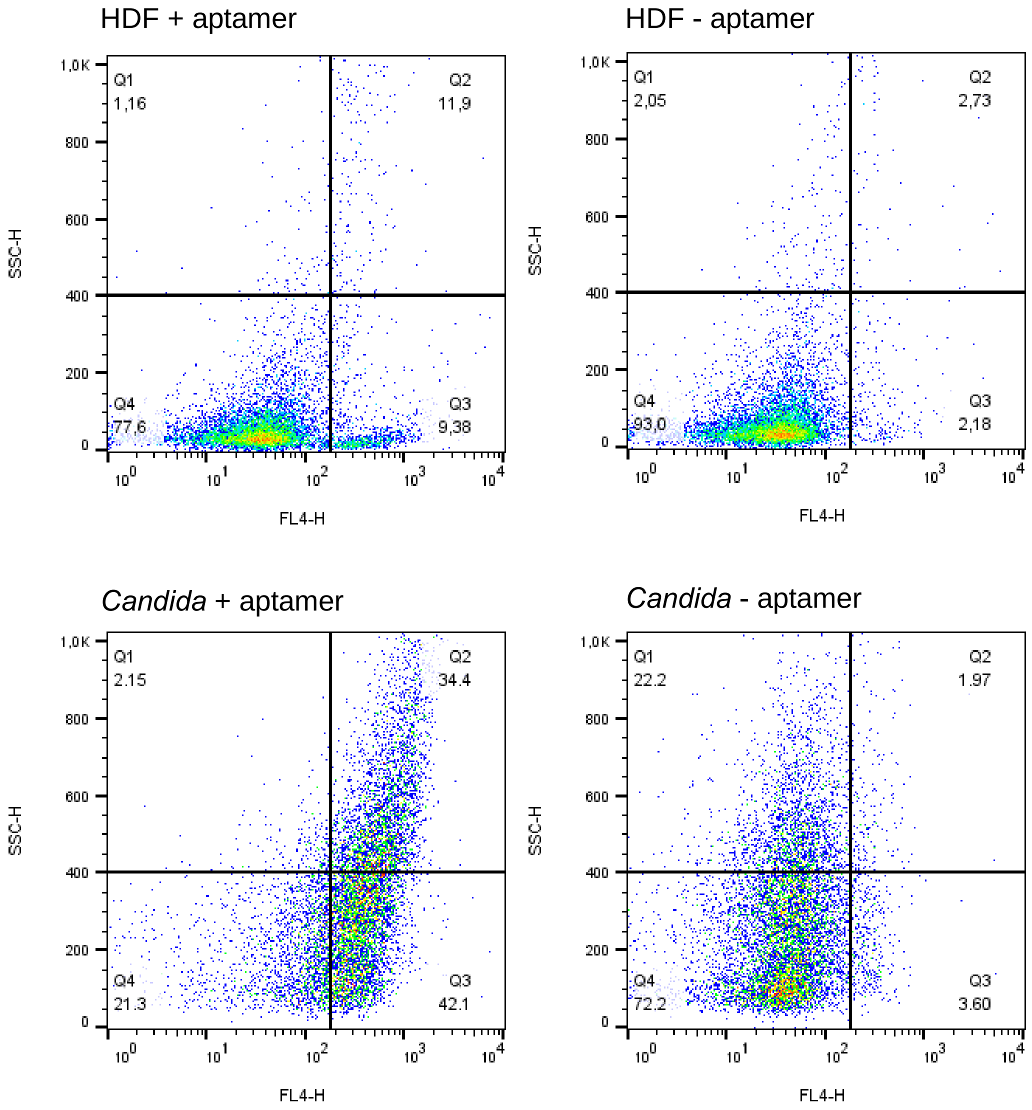

Supplementary Figure S1. Flow-cytometry data show detection of fluorescently labeled *Candida* cells. 10,000 cells were incubated with 10 pmol aptamers in 250 µL PBS buffer, incubated for 15 minutes at 37 °C and then measured with a FACScalibur (BD, Franklin Lakes, USA).
